# A catalytically and genetically optimized β-lactamase-matrix based assay for sensitive, specific, and higher throughput analysis of native henipavirus entry characteristics

**DOI:** 10.1186/1743-422X-6-119

**Published:** 2009-07-31

**Authors:** Mike C Wolf, Yao Wang, Alexander N Freiberg, Hector C Aguilar, Michael R Holbrook, Benhur Lee

**Affiliations:** 1 Department of Microbiology, Immunology, and Molecular Genetics, UCLA, Los Angeles, CA, USA 90095; 2 Department of Pathology and Laboratory Medicine, UCLA, Los Angeles, CA, USA 90095; 3 UCLA AIDS Institute, UCLA, Los Angeles, CA, USA 90095; 4 Department of Pathology, University of Texas, Medical Branch, UTMB, Galveston, TX, USA 77555

## Abstract

Nipah virus (NiV) and Hendra virus (HeV) are the only paramyxoviruses requiring Biosafety Level 4 (BSL-4) containment. Thus, study of henipavirus entry at less than BSL-4 conditions necessitates the use of cell-cell fusion or pseudotyped reporter virus assays. Yet, these surrogate assays may not fully emulate the biological properties unique to the virus being studied. Thus, we developed a henipaviral entry assay based on a β-lactamase-Nipah Matrix (βla-M) fusion protein. We first codon-optimized the bacterial β*la *and the *NiV-M *genes to ensure efficient expression in mammalian cells. The βla-M construct was able to bud and form virus-like particles (VLPs) that morphologically resembled paramyxoviruses. βla-M efficiently incorporated both NiV and HeV fusion and attachment glycoproteins. Entry of these VLPs was detected by cytosolic delivery of βla-M, resulting in enzymatic and fluorescent conversion of the pre-loaded CCF2-AM substrate. Soluble henipavirus receptors (ephrinB2) or antibodies against the F and/or G proteins blocked VLP entry. Additionally, a Y105W mutation engineered into the catalytic site of βla increased the sensitivity of our βla-M based infection assays by 2-fold. *In toto*, these methods will provide a more biologically relevant assay for studying henipavirus entry at less than BSL-4 conditions.

## Background

The henipaviruses, Nipah (NiV) and Hendra (HeV), are emerging zoonoses; the former caused multiple outbreaks of fatal encephalitis in Malaysia, Bangladesh, and India with mortalities ranging from 40-70% while the latter produced respiratory syndromes among thoroughbred horses in Australia whilst also being implicated in the death of a horse handler [1-4]. These two paramyxoviruses, both designated Category C priority pathogens by the NIAID Biodefense Research Agenda, require strict Biosafety Level 4 (BSL-4) containment due to their extreme pathogenicity, unverified mode(s) of transmission, and lack of pre- or post-exposure treatments[4].

BSL-4 containment limits the opportunities for thorough analysis of live henipavirus entry characteristics. Surrogate assays to study henipavirus entry at less than BSL-4 conditions exist, such as cell-cell fusion or VSV-based NiV-envelope pseudotyped reporter assays. These assays have been used to probe envelope receptor interactions and characterize the determinants of fusion with regards to both the fusion (F) and attachment (G) envelope glycoproteins [5-10]. However, cell-cell fusion lacks the geometric and kinetic constraints found in virus-cell fusion while pseudotyped VSV particles physically resemble *Rhabdoviridae *rather than the pleomorphic *Paramyxoviridae*. Therefore, neither assay may fully recapitulate the biological properties of native envelope structures of live henipaviruses. Moreover, pseudotype reporter virus assays depend on efficient transcription and translation of a reporter gene after virus entry. Thus, earlier steps in viral entry, such as matrix uncoating, may also not be resolved by either of these assays.

Many viruses form virus-like particles (VLPs) via expression of their matrix alone (*e.g. *Sendai, HPIV-1, Ebola, HIV, Rabies) or only in combination with envelope proteins (*e.g. *Simian Virus 5, Measles) [11-19]. Paramyxoviral matrix proteins direct budding of virions from the surface of infected cells and interact with the endodomain of envelope proteins, ultimately assisting in viral assembly[11,20]. Specifically, NiV matrix (NiV-M) alone, or in combination with its fusion protein (NiV-F) and receptor-binding protein (NiV-G), buds and forms VLPs efficiently[21,22]. Additionally, matrix may function to recruit the nucleoprotein-encased genome to the budding site[15,23]. Paramyxoviral matrix proteins perform essential roles in viral release/budding and presumably rely on late domains[20,24] for these functions; although typical late domain motifs have not been found in certain paramyxoviral M proteins[25]. Thus, NiV matrix-based VLPs will likely better reflect the biological properties of their live-virus counterparts in entry assays. Here, we developed a VLP-based assay that can be used for analyses of henipaviral entry characteristics under BSL-2 conditions. This VLP assay is based on a β-lactamase-Nipah Matrix (βla-M) fusion reporter protein.

β-lactamase (βla) is a commonly used reporter protein whose reporter activity depends on its ability to cleave β-lactam ring-containing fluorescent or colorimetric substrates. For our purposes, CCF2-AM proved useful as a cell-permeant fluorescent substrate engineered to exhibit a shift from green to blue fluorescence upon βla cleavage [26-28]. CCF2-AM cell loading is nearly 100% efficient, practically irreversible (cytoplasmic esterases prevent CCF2 from diffusing out of the cells), and permits loading of a variety of cell types including primary neuron or microvascular endothelial cells, the main targets of NiV infection. Thus, virus-cell fusion of envelope bearing βla-M VLPs should deliver βla-M to the cytosol leading to fluorescent conversion of the pre-loaded CCF2 substrate. The shift from green to blue fluorescence can then be quantified by flow cytometry or quantitative microscopy.

Genetic optimization of both the expression and the intrinsic enzymatic efficiency of the βla-M reporter allowed for sensitive, specific and relatively high-throughput analyses of henipavirus entry in the absence of vaccinia augmentation. Our results suggest that this strategy may be generalized to other viruses where matrix is the primary determinant of budding and virion morphology.

## Results

### Synthesis of the β-lactamase-Nipah Matrix (βla-M) fusion construct and its incorporation into virus-like particles (VLPs)

**Figure 1:**
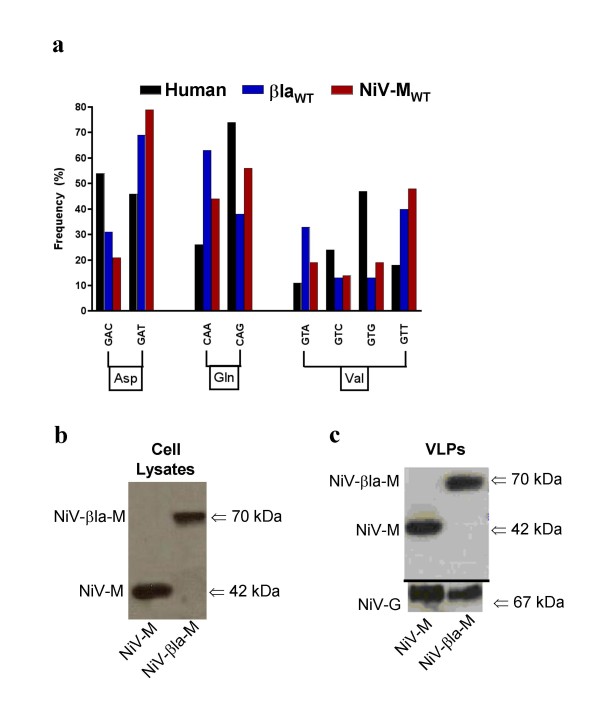
**Synthesis of the β-lactamase-matrix (βla-M) fusion construct and its incorporation into virus-like particles (VLPs)**. **a) **Codon usage comparisons between wild-type *NiV-M *(henipavirus), β*la *(bacteria) and average Homo sapiens genes. For clarity, only representative amino acids with significant differences in codon usage frequencies between Homo sapiens and *NiV-M *or β*la *genes are shown. Note the skewing towards more rarely used mammalian codons. Overall, codon usage for amino acids not shown cumulatively demonstrate a pattern of rare mammalian codon usage (see Additional file 1). **b) **Cell lysates from transfected 293T cells were blotted for protein expression using anti-M antibodies. **c) **VLPs collected from NiV-M+NiV-F/G or βla-M+NiV-F/G transfected 293T cell supernatants were purified as described in the materials and methods. VLPs were lysed and blotted for protein incorporation using anti-NiV-M antibodies along with anti-HA (NiV-G) antibodies to quantify total VLP production.

NiV-M is a small, basic and moderately hydrophobic 352 amino-acid protein and one of the most abundant proteins within the virion. Therefore, we chose to fuse a reporter protein to NiV-M in a manner that does not interfere with its ability to form VLPs. Published data shows that the C-terminal end of many matrix proteins regulates complex functions involved in budding and viral assembly[20,25,29-35]; thus, we decided to fuse the β-*lactamase *gene (β*la*) onto the N-terminus of *NiV-M*. Examination of the codon-usage of wild-type β*la *and wild-type *NiV-M *revealed a skewing towards the use of rare mammalian codons (Fig. 1a). Therefore, we codon-optimized both β*la *and *NiV-M *to produce a fully codon-optimized β*la-M *gene for efficient expression in mammalian cells (see **Materials and Methods**).

Codon-optimized NiV-M and βla-M were equivalently expressed in transfected 293T cells (Fig. 1b). Notably, fusion of codon-optimized β*la *to wild-type *NiV-M *(*NiV-M*_*WT*_) resulted in almost undetectable expression of βla-M under similar transfection conditions (data not shown). To verify incorporation of NiV-M and βla-M into VLPs, we transfected 293T cells with codon-optimized NiV-M or βla-M along with the corresponding codon-optimized NiV-F and NiV-G envelope glycoproteins. After isolating VLPs from the transfected cell supernatants, we verified the presence of NiV-M or βla-M within the lysed VLPs by immunoblotting with NiV-M-specific antibodies (Fig. 1c). Only M-containing VLPs with both NiV-F and NiV-G on their surface will be infectious in our entry assays and these data suggest that fusion of βla to NiV-M did not perturb the ability of NiV-M to form VLPs or incorporate cognate viral envelope glycoproteins. Coexpression of nucleocapsid (N) along with NiV-M or βla-M did not alter the overall production of M-containing VLPs (data not shown), consistent with findings from other groups[21].

### βla-M+NiV-F/G VLPs morphologically, biochemically, and biologically mimic live NiV

**Figure 2:**
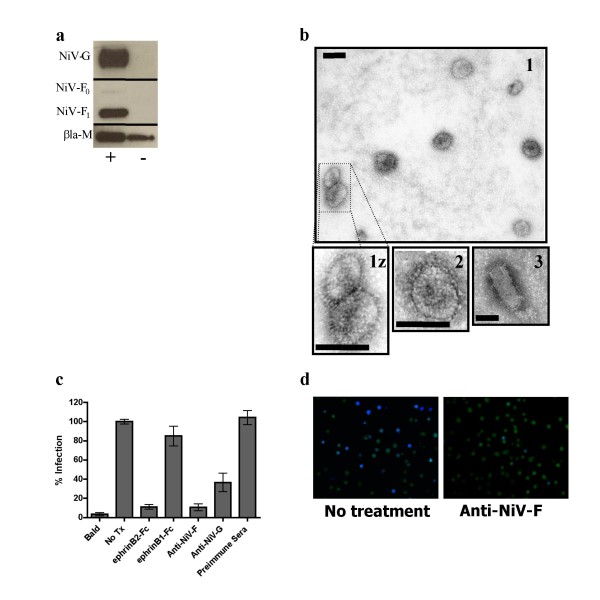
**βla-M+NiV-F/G VLPs morphologically, biochemically, and biologically mimic live NiV**. **a) **VLPs produced in the presence (**+**) or absence (-) of envelope proteins were lysed and blotted for protein incorporation using anti-HA (NiV-G), anti-AU1 (NiV-F), or anti-NiV-M antibodies. **b) **Purified particles were analyzed under electron microscopy as described in materials and methods at 72,000× magnification. 1(z) = βla-M+NiV-F/G VLPs, 2 = NiV-M+F/G VLPs, 3 = pseudotyped VSV+NiV-F/G. Scale bars represent 100 nm. **c) **Vero cells were infected with NiV-F/G VLPs containing the βla-M fusion protein. Soluble ephrinB2-Fc and ephrinB1-Fc were added to a final concentration of 75 nM. Anti-NiV-F (834), anti-NiV-G (806), and pre-immune sera were added to a final concentration of 5 μg/ml. Infected cells (% blue positive) were quantified using flow cytometry with untreated entry (NoTx) normalized as 100%. Data shown as an average of triplicates from three individual experiments ± SEM. **d) **Fluorescence microscopy was performed on representative corresponding wells from (**c) **at 20× magnification using a beta-lactamase dual-wavelength filter (Chroma Technologies, Santa Fe Springs, CA).

NiV-M will bud and form VLPs in the presence or absence of co-transfected NiV-F and NiV-G[21,22]. Thus, we also determined how well βla-M would bud and form VLPs in the presence or absence of NiV-F and NiV-G. Fig. 2a shows that the βla-M construct also budded and formed VLPs in the presence and absence of the NiV envelope proteins, similar to what has been shown for NiV-M[21,22].

Next, we characterized the morphology of the VLPs by imaging the βla-M VLPs via electron microscopy. Fig. 2b shows that βla-M VLPs closely resembled the morphology and size of standard NiV-M VLPs, and both exhibited the standard pleomorphic shape representative of *Paramyxoviridae*, ranging in size from 50 nm to 800 nm[36]. The images also resolved the presence of viral "spikes" protruding from the particles; these represent the viral envelope glycoproteins of NiV on the surface of the particle, confirming their incorporation into the VLPs. Tellingly, pseudotyped VSV+NiV-F/G particles resembled classical bullet-shaped *Rhabdoviridae *particles (Fig. 2b). This further underscores potential biological differences that may occur when using NiV-M based VLPs versus VSV pseudotypes.

Fig. 2c shows the specificity and sensitivity of our βla-M VLP entry assay via flow cytometry analyses. Entry of βla-M+NiV-F/G VLPs into Vero cells produced signals with a 25-fold dynamic range over βla-M VLPs lacking NiV viral envelope proteins (Fig. 2c). For simplicity, we will refer to successful entry of βla-M+NiV-F/G VLPs into susceptible cells as "infection" and to βla-M VLPs lacking NiV viral envelope proteins as "bald" VLPs. To verify receptor-specificity within our assay, we infected in the presence of soluble NiV receptor, ephrinB2-Fc, which successfully inhibited infection while a non-receptor homologue, ephrinB1-Fc, did not (Fig. 2c). In addition, anti-NiV-F and anti-NiV-G polyclonal antibodies[10,37], but not the pre-immune sera, also inhibited infection (Fig. 2c) emphasizing that the βla-M+NiV-F/G VLPs emulate the known roles of F and G in mediating paramyxoviral entry. Green to blue color shifts in CCF2-loaded cells were also confirmed visually (Fig. 2d) before flow analyses. Collectively, these data establish that the βla-M VLPs physically and biochemically resemble NiV while the infection reflects the receptor and envelope specificity of live Nipah viruses.

### βla-M+NiV-F/G VLPs infect biologically relevant cells in a receptor-dependent manner

**Figure 3:**
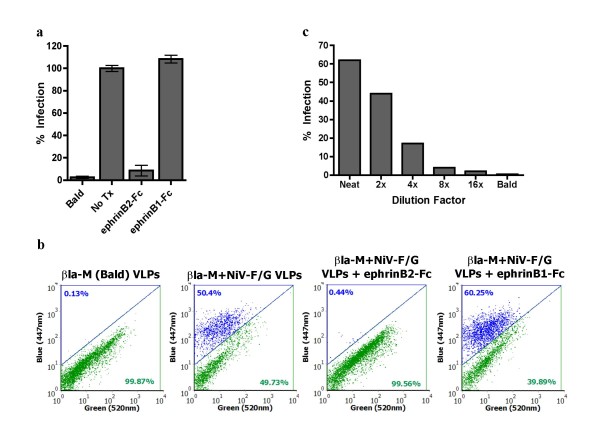
**βla-M+NiV-F/G VLPs infect a biologically relevant cell line in a receptor-dependent manner**. **a) **HMVECs were infected with βla-M+NiV-F/G or βla-M-only VLPs and quantified via flow cytometry. Soluble ephrinB2-Fc or ephrinB1-Fc was added at a final concentration of 75 nM. Infected cells (% blue positive) were quantified using flow cytometry with untreated entry (NoTx) normalized as 100%. Data shown as an average of triplicates from three individual experiments ± SEM. **b) **Representative flow cytometry plots of the data from **(3a)**. **c) **βla-M+NiV-F/G VLPs from **(a) **were diluted in increments and used to infect HMVECs as previously described. Infected cells (% blue positive) were quantified using flow cytometry. Data shown as singlets from a single representative experiment.

To further illustrate the biological relevance of our βla-M VLP entry assay, we used βla-M VLPs to infect primary cell targets of natural NiV infection. The formation of giant-multinucleated syncytia in human microvascular endothelial cells (HMVECs) is a pathogenic hallmark of NiV infection[38]. Thus, we used βla-M VLPs to infect HMVECs preloaded with CCF2-AM (Fig. 3a and Fig. 3b). Interestingly, we observed a significant improvement in signal to noise ratio compared to the read-out from Vero cell infections. Again, the cognate soluble NiV receptor, ephrinB2-Fc, but not ephrinB1-Fc, inhibited infection of HMVECs, underscoring the receptor specificity of NiV VLP infection in these primary cells (Fig. 3a and Fig. 3b). Finally, to demonstrate that these infections took place within the linear range of our assay, we serially diluted the βla-M VLPs as indicated and found the amounts used to infect HMVECs were within the linear range (Fig. 3c).

### Hendra virus (HeV) envelope proteins package efficiently onto βla-M(NiV) and produce infectious VLPs

**Figure 4:**
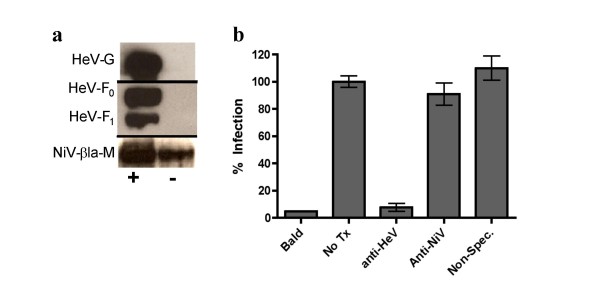
**Hendra virus (HeV) envelope proteins package efficiently onto βla-M(NiV) and produce infectious VLPs**. **a) **VLPs collected from βla-M(NiV)+ HeV-F/G or βla-M(NiV)-only transfected 293T supernatant were purified as described in the materials and methods. VLPs were lysed and blotted for proteins using anti-HA (HeV-G), anti-AU1 (HeV-F), or anti-NiV-M antibodies. **b) **HMVECs were infected by βla-M(NiV)+ HeV-F/G VLPs in the presence of anti-HeV-F specific (mAb 36) or anti-NiV-F specific (mAb 66)[37] monoclonal antibodies with non-specific monoclonal antibodies as a negative control to a final concentration of 20 μg/ml. Infected cells (% blue positive) were quantified using flow cytometry with untreated (NoTx) entry normalized as 100%. Data shown as an average of singlets from three individual experiments ± SD.

Molecular and immunological data indicate that NiV and HeV are closely related viruses that can be appropriately clustered into a new henipavirus genus. Indeed, NiV and HeV F and G proteins can functionally cross-complement each other[5,39]. However, it remains unknown whether NiV-M can complement the function of HeV-M to produce infectious HeV envelope bearing VLPs. While rhabdoviral matrices can functionally accommodate many heterologous envelope proteins, it is less clear whether paramyxoviral matrix proteins can incorporate heterologous envelope proteins in a functional manner. Fig. 4a shows that our βla-M(NiV) construct allowed efficient formation of HeV-enveloped VLPs at levels equivalent to NiV-enveloped VLPs (Fig. 4a and 2a). Infecting HMVECs with βla-M(NiV)+HeV-F/G VLPs produced a similar dynamic range to that of βla-M(NiV)+NiV-F/G particles (data not shown). βla-M(NiV)+HeV-F/G VLP infection was similarly envelope dependent as an anti-HeV-F specific monoclonal antibody inhibited infection while an anti-NiV-F specific monoclonal[37] and non-specific monoclonal antibodies had little to no effect (Fig. 4b).

### βla-M VLPs enveloped with the NiV-G_E505A _mutant recapitulate differential receptor usage

**Figure 5:**
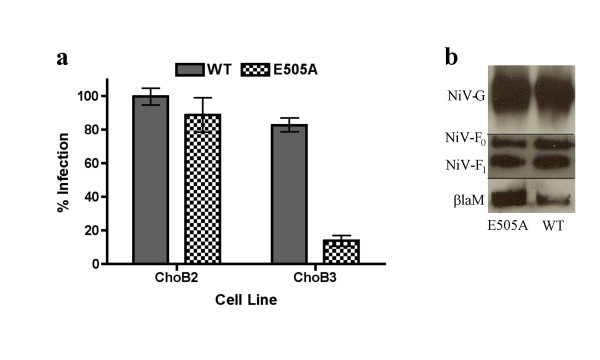
**βla-M VLPs enveloped with the NiV-G_E505A _mutant recapitulate differential receptor usage**. **a) **Enveloped βla-M VLPs incorporating an E505A mutation in NiV-G were used to infect CHO-B2 or CHO-B3 cells stably expressing only ephrin-B2 or ephrin-B3, respectively. Infected cells (% blue positive) were quantified using flow cytometry with ephrin-B2 mediated entry normalized as 100%. Data shown as an average of triplicates from three individual experiments ± SEM. **b) **VLPs from **(5a) **were lysed and blotted for protein incorporation using anti-HA (NiV-G/NiV-G_E505A_), anti-AU1 (NiV-F), or anti-NiV-M antibodies.

NiV and HeV exhibit analogous tropisms and both utilize ephrinB2 and ephrinB3 for cellular entry; although how well ephrinB2 or ephrinB3 allows for entry into various primary cell targets of henipavirus infections remains to be defined[9,40]. However, both NiV and HeV utilize ephrinB2 with much greater efficiency than ephrinB3[9,40]. Interestingly, a point mutation (E505A) within the globular domain of NiV-G abrogates efficient B3-dependent entry while leaving B2-dependent entry unaffected[39]. We previously argued that differential ephrinB2 versus B3 usage may have direct pathogenic relevance as *only *ephrinB3 is expressed in the brainstem[39,41], the site of neuronal dysfunction ultimately causing death from encephalitis after NiV infection[42]. Thus, to fully contextualize this previously reported phenotype, we sought to determine if the differential receptor usage of the NiV-G_E505A _mutant is fully recapitulated using βla-M VLPs. Indeed, incorporation of an NiV-G_E505A _envelope mutant along with NiV-F onto βla-M resulted in VLPs defective in their ability to gain entry into CHO-B3 cells, but not CHO-B2 cells (Fig. 5a)[39]. Fig. 5b shows that both the NiV-G_E505A _mutant and NiV-G_WT _(both along with NiV-F) are equivalently incorporated into VLPs and, thus, the differential receptor usage phenotype was not due to different levels of envelope incorporation.

### A Y105W mutation within the active site of βla increases cleavage efficiency resulting in a more sensitive entry assay

**Figure 6:**
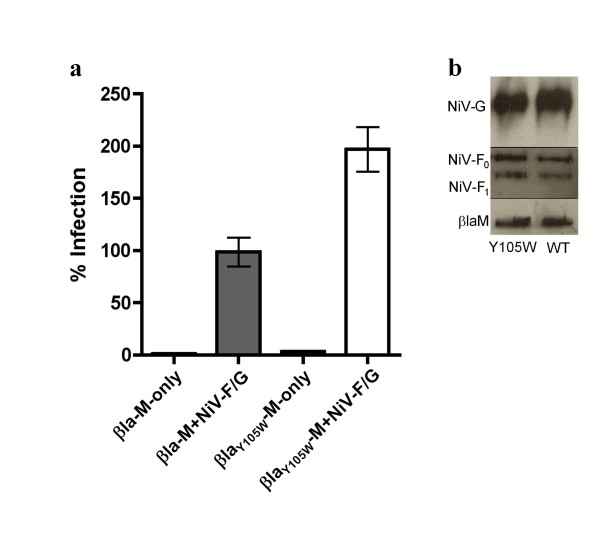
**A single amino acid (Y105W) mutation within the active site of βla increases cleavage efficiency resulting in a more sensitive entry assay**. **a) **Vero cells were infected with βla-M, βla_Y105W_-M, βla-M+NiV-F/G and βla_Y105W_-M+NiV-F/G VLPs. Infected cells (% blue positive) were quantified using flow cytometry with βla-M+NiV-F/G infection normalized as 100%. Data shown as an average of triplicates from one representative experiment ± SD. **b) **VLPs were lysed and blotted for protein incorporation using anti-HA (NiV-G), anti-AU1 (NiV-F), and anti-NiV-M antibodies.

To further increase the sensitivity of our βla-M based assay for future high-throughput tasks, we sought to improve the catalytic activity of βla. Active site mutations have been shown to increase the substrate cleavage efficiency of βla for certain β-lactam containing antibiotics in an enzyme subtype and substrate specific manner [43-46]. Thus, we searched the literature for active site mutations that increase the catalytic activity of the βla (TEM1 strain) for the substrate cefazolin, the most closely related β-lactam to CCF2-AM. A tyrosine to tryptophan (Y105W) mutation within the active site of the TEM1-βla increases the catalytic activity (K_cat_/K_m_) for cefazolin by 1.5-fold[46]. Therefore, we engineered this Y105W mutation into βla-M (βla_Y105W_-M) in order to increase the assay sensitivity and make the system more amenable to high-throughput tasks. Indeed, βla_Y105W_-M increased the signal to noise ratio obtained in our VLP entry assay 1.8-fold (Fig. 6a) while overall VLP production levels remained similar (Fig. 6b). Thus, βla_Y105W_-M appears to have increased the sensitivity of our VLP entry assay on a per virion basis.

## Discussion and conclusion

Many viral entry studies on highly pathogenic agents have relied on cell-cell fusion and envelope pseudotyped reporter assays which have permitted detailed analyses of their entry characteristics without high-level biosafety containment. Yet, these surrogate assays may not fully emulate the biological properties unique to the virus being studied. Cell-cell fusion assays do not mimic virus-cell fusion kinetics and are not constrained by the geometry of virus-cell fusion, and envelope pseudotyped viral systems reflect the virion morphology of the backbone virus rather than the parental virus from which the envelopes are derived. Such differences may confound accurate dissection of the entry pathway under study. Pseudotyped reporter virus assays also require efficient replication and transcription of the reporter gene in the cell type used, and thus, post-entry factors may influence the efficiency of reporter gene expression. For BSL-4 containment viruses like NiV and HeV, the problems are compounded by the limited availability of resources to confirm the results of surrogate assays in live henipaviruses. Thus, we sought to develop a system that more faithfully replicates the native henipavirus entry process. This will allow for a more detailed and biologically relevant analysis of early entry events and will facilitate the development of high-throughput screens for inhibitors of *bona fide *henipavirus entry processes.

VLPs can be produced via expression of viral matrices alone or in combination with their respective envelope proteins [11-19]. Paramyxoviral matrix proteins, abundant within the virion, seemingly act as the 'bandleader' by coordinating several events within the viral life cycle: envelope protein localization, assembly and budding, nucleocapsid or genome recruitment, and particle disassembly or uncoating[11,47]. Thus, these VLPs more faithfully mimic their live virus counterparts and permit a more biologically relevant analysis of entry and uncoating kinetics. Despite these many functionalities, none appear to be significantly disrupted by fusing large reporter proteins like GFP, *Renilla *luciferase, or βla to the N-terminus of NiV-M[22] (Fig. 2 and unpublished observations). Thus, we sought to exploit this property by fusing the β-lactamase enzyme to the N-terminus of NiV-M in an effort to create a sensitive and specific viral entry assay.

Several viral entry assays have been developed that rely on cytosolic delivery, or intravirion detection, of a virion associated reporter fusion protein. For example, entry assays using vpr-βla for HIV and βla-matrix for Ebola have been described[48,49], yet the published assays would appear to be less sensitive than our current system[48,50]. In the process of making our βla-M reporter, we discovered that both the *NiV-M *and the β*la *genes tended to use rare mammalian codons (Fig. 1a and see Additional file 1). Indeed, our β*la-M *fusion construct yielded significant protein expression only when both genes were fully codon-optimized (Fig. 1b-c and data not shown). This could explain why NiV-M is poorly expressed in the absence of vaccinia augmentation[21] and why βla based real-time fusion assays are more sensitive and robust when using codon-optimized βla[37]. Codon-optimization alone likely results in the larger dynamic range and greater sensitivity of our βla-M based assays.

Our βla-M VLPs adopt the pleomorphic morphology of paramyxoviruses and incorporate henipaviral envelopes in a manner indistinguishable from wild-type NiV-M VLPs. NiV and HeV envelope bearing βla-M VLPs recapitulate their biological phenotypes in terms of receptor usage and the requirements for F and G in the paramyxoviral entry process (Figs. 2, 3, 4 and 5). Importantly, βla-M VLPs can be used to study early entry events in primary cell targets of henipavirus infections, such as HMVECS, without potentially confounding factors like virus replication mediated cytotoxicity or other post-entry restriction factors. Significantly, the βla-M VLPs can also assay virus uncoating (*i.e. *virus-cell content mixing) via detection of viral matrix protein exposure to the cellular cytoplasm.

While it is clear that *Rhabdoviridae *can functionally accommodate many different heterologous envelopes [51-54], it is less clear whether paramyxoviral matrix proteins have the ability to functionally cross-complement other members of the family. We demonstrated here that βla-M(NiV) was able to complement and package the HeV envelope proteins, emphasizing the relatedness between these two viruses. Our results open the possibility that other paramyxoviral envelope proteins can functionally cross-complement onto βla-M(NiV), or their own respective βla-matrix fusion constructs, thereby providing a more efficient and high-throughput assay to study paramyxoviral entry. Arguably, short of reverse genetics to study matrix and envelope mutants in the context of parent paramyxoviruses, this βla-M VLP assay better reflects the native biology of paramyxoviral entry than other surrogate assays. To further improve the sensitivity of this assay for high-throughput applications, we exploited the vast literature on β-lactam structure-function studies and engineered a Y105W mutation into the active site of βla known to increase the cleavage efficiency of the enzyme [43-46] (Fig. 6).

In summary, we have developed a codon-optimized catalytically improved βla-M based VLP system that can be used for henipaviral entry studies. The flexibility of using either colorimetric or cell permeant fluorimetric substrates in the same βla-M VLP system allows for efficient, quantitative, and more high throughput analyses of henipavirus fusion and entry characteristics that more closely reflect those of authentic viral particles. Whether βla-M can be complemented with other paramyxoviral envelopes remains to be determined, but such studies will provide information into the specificity of matrix-envelope interactions. Lastly, our results imply that such a codon-optimized, catalytically improved βla-M based entry system may be adapted to other viruses that possess a matrix protein primarily responsible for virion morphology and budding characteristics.

## Materials and methods

### Codon optimization and expression plasmids

The codon-optimized NiV-F or HeV-F and NiV-G or HeV-G gene products were tagged at their C-termini with an AU1 or hemagglutinin (HA) tag, respectively, as previously described[37,39]. *NiV-M*_*WT *_was synthesized by Origene (Rockville, MD). GeneArt (Regensburg, Germany) performed mammalian codon-optimization of the *NiV-M *gene (M) product according to in-house proprietary software that addresses codon usage, elimination of cryptic splicing sites, as well as the stability of DNA/RNA secondary structures. *NiV-M *was subcloned into pcDNA3.1 (Invitrogen, Carlsbad, CA) between HindIII and XhoI restriction enzyme sites. The sequence of the codon-optimized *NiV-M *has been deposited into GenBank (Accession: EU480491). Origene (Rockville, MD) codon-optimized the β*la *gene, which was then subcloned into a pVAX1 (Invitrogen) expression vector between the KpnI and XhoI restriction enzyme sites. The sequence of the mammalian codon-optimized β*la *has been deposited into GenBank (Accession: EU744548). The β*la *gene was fused upstream of the *NiV-M *gene by overlap PCR and subsequently cloned into pcDNA3.1 via flanking KpnI and XhoI restriction enzyme sites with a NotI restriction enzyme site engineered in between the two genes. A single Y105W amino acid mutation within the βla active site was introduced using site-directed mutagenesis with QuikChange™ (Stratagene, La Jolla, CA). β*la*_*Y*105*W *_was then cloned into pcDNA3.1 via flanking KpnI and NotI restriction enzyme sites. All gene products were confirmed by sequencing.

### Antibody Production

Production protocols to provide polyclonal antibodies (Rb. #2702, terminal bleed) via immunized rabbits (using a 20-mer antigenic peptide sequence corresponding to amino acids 29-49 of NiV-M) were generated by the Pinnacle Antibody Program (21^st ^Century Biochemicals, Marlboro, MA). Monoclonal anti-HeV specific antibodies were produced by expressing HeV-F, HeV-G, and NiV-M in rabbits then isolating and screening specific anti-HeV lymphocytes from rabbit spleens as previously described for anti-NiV-F specific monoclonal antibodies[37].

### Cell culture

293T cells were grown in Dulbecco's modified Eagle's medium (Invitrogen) containing 10% fetal bovine serum (FBS) (Omega Scientific, Tarzana, CA). Vero cells were grown in minimal essential medium alpha (Invitrogen), containing 10% FBS. CHO stable cell lines expressing ephrinB2 or ephrinB3 were derived and maintained as previously described[9]. HMVECs were grown in EGM-2 media supplemented with the MV Bullet Kit (Cambrex, Baltimore, MD). 293T and Vero cells were purchased from the ATCC. HMVEC cells were a kind gift from R. Shao.

### Production of βla-M(NiV) VLPs

β*la-M *expression plasmids (25 μg) and either *NiV-F *and *G *or *HeV-F *and *G *(10 μg each) or *pcDNA3 *(20 μg) expression plasmids were transfected into 10 cm dishes of 293T cells using Lipofectamine 2000 (Invitrogen). At 24 h post-transfection, supernatants were collected and clarified before pelleting the VLPs at 110,000 *g *through a 20% sucrose (in PBS) cushion followed by resuspension in PBS (Invitrogen) containing 5% sucrose.

### Immunoblotting of VLP proteins

βla-M VLP-containing supernatants were lysed and separated by sodium dodecyl sulfate-polyacrylamide gel electrophoresis (SDS-PAGE) and subsequently detected by immunoblotting using rabbit-anti-NiV-matrix (to detect all NiV-M proteins), goat-anti-HA-HRP (to detect all G proteins) (Novus Biologicals, Littleton, CO), or mouse-anti-AU1 (to detect all F proteins) (Covance, Princeton, NJ) antibodies. Primary and secondary antibodies were used at 1:1,000 and 1:80,000 dilutions, respectively, or 1:10,000 for anti-HA-HRP followed by FEMTO (Pierce, Rockford, IL) detection. Due to the similar molecular weights of βla-M (~70 kDa) and NiV-G (~67 kDa), membranes were probed for NiV-M, NiV-F or HeV-F, and NiV-G or HeV-G individually.

### Electron microscopy

200-mesh Formvar carbon-coated copper grids (Electron Microscopy Sciences, Hatfield, PA) were floated on drops of the NiV VLP suspensions at room temperature, then blotted and stained with 1% aqueous uranyl acetate (UA) for NiV VLPs and 2% aqueous solution of phosphotungstic acid (PTA) for VSV particles. Electron microscopy studies were performed on a Philips 201 electron microscope at 70 kV.

### Quantification of βla-M VLP entry via FACS Aria

Cells were plated into 24-well plates at a confluency of 75% and spinoculated (2,000 *g*) with βla-M VLPs for 2 h at 37°C. Although not required for efficient VLP entry, spinoculation has been shown to significantly improve the entry efficiency of several viruses (e.g. HIV, HHV-6, CMV) into target cells[55,56] and, indeed, improved the signal to noise ratio within our assay (data not shown). Target cells were then stained with CCF2-AM substrate according to the manufacturer recommendations (Panvera, Madison, WI). The enzymatic reaction was allowed to take place at 25°C for 18 h. The cells were then washed, resuspended in FACS-buffer (2% FBS in PBS) and fixed with 2% paraformaldehyde. Cells were then acquired using FACS-Diva software on a FACS Aria machine (BD Biosciences, San Diego, CA) with excitation at 407 nm and emission at 520 nm and 447 nm. Samples were analyzed using FACS Convert and FCS Express v3 (De Novo Software, Los Angeles, CA). Soluble ephrinB1-Fc and ephrinB2-Fc fusion proteins were purchased from R&D Systems (Minneapolis, MN). Data were analyzed by GraphPad™ Prism Software (San Diego, CA) and represented as percentage infection (% blue positive cells).

## Competing interests

The authors declare that they have no competing interests.

## Authors' contributions

MCW carried out or took part in all experiments, participated in the design and coordination of the study, performed statistical analyses, and wrote the manuscript. YW assisted with Western blot analyses and proofread the manuscript. ANF assisted with electron microscopy studies and proofread the manuscript. HCA assisted with antibody competition studies. MRH coordinated portions of the study, proofread the manuscript, and supervised electron microscopy studies. BL conceived the study, participated in its design and coordination, and helped draft the manuscript. All authors read and approved the final manuscript.

## Supplementary Material

Additional file 1 **Comparative codon usage table**. Codon usage comparisons between wild-type Nipah matrix (henipavirus), beta-lactamase (bacteria) and average Homo sapiens genes. Click here for file
