# Supplementary material for: A catalytically and genetically optimized β-lactamase-matrix based assay for sensitive, specific, and higher throughput analysis of native henipavirus entry characteristics
Source: Virol J. 2009 Jul 31;6:119. doi: 10.1186/1743-422X-6-119 (PMC2727953; doi:10.1186/1743-422X-6-119)
Supplement: Additional file 1 — Comparative codon usage table. Codon usage comparisons between wild-type Nipah matrix (henipavirus), beta-lactamase (bacteria) and average Homo sapiens genes. [file 1743-422X-6-119-S1.pdf]

Additional file 1

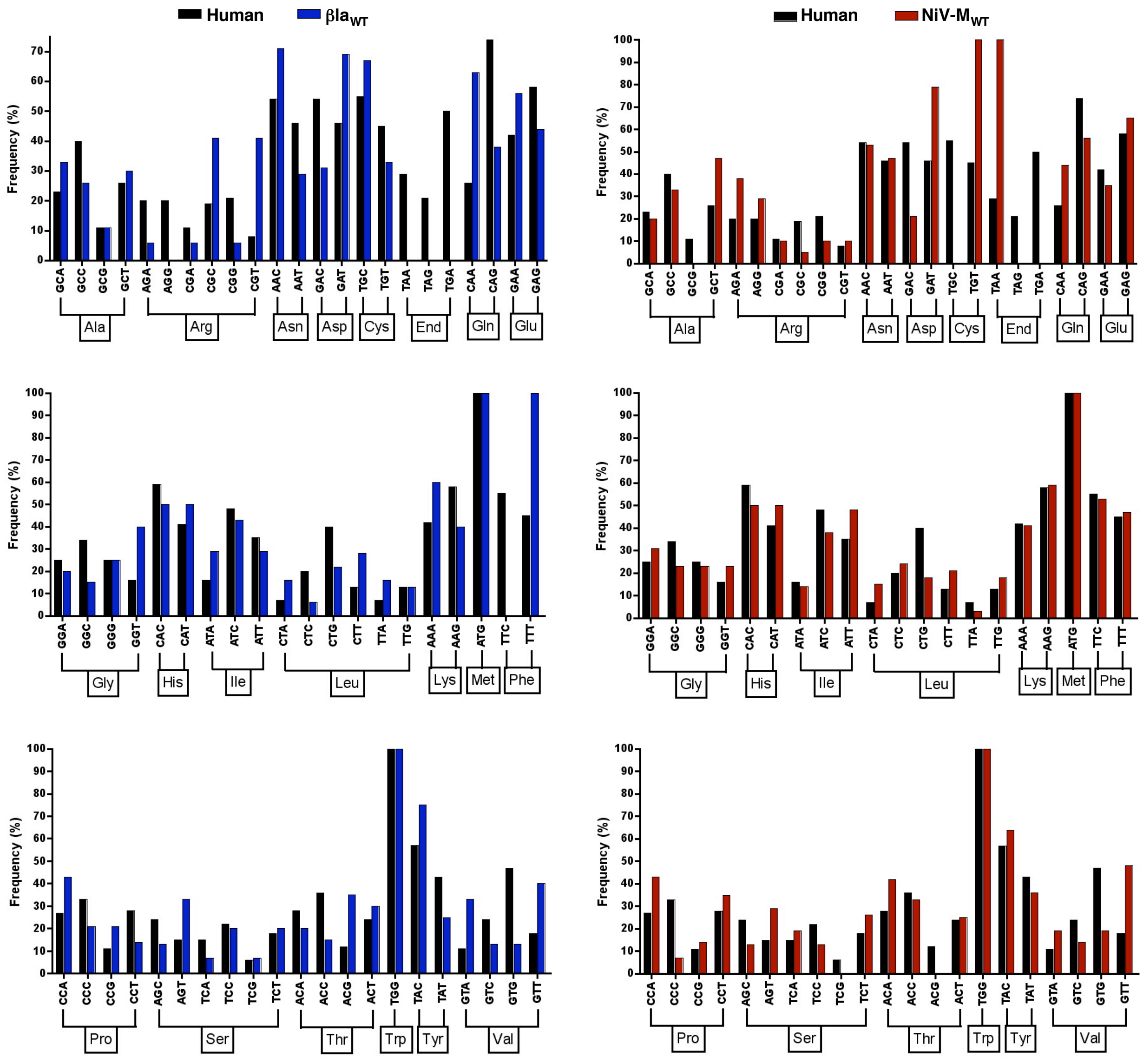

Table 1. Codon usage comparisons between wild-type Nipah matrix (henipavirus), beta-lactamase (bacteria) and average Homo sapiens genes. Note the skewing towards more rarely used mammalian codons. Overall, codon usage for amino acids cumulatively demonstrates a pattern of rare mammalian codon usage.
